# Supplementary material for: Assessment of Tenderness and Anthocyanin Content in Zijuan Tea Fresh Leaves Using Near-Infrared Spectroscopy Fused with Visual Features
Source: Foods. 2025 Aug 22;14(17):2938. doi: 10.3390/foods14172938 (PMC12428271; doi:10.3390/foods14172938)
Supplement: Supplementary file 1 [file foods-14-02938-s001.zip › foods-3786596-supplementary.pdf]

Supplementary Material

Table S1. The content of trace anthocyanins in different tenderness grades.

Anthocyanin	Level	Range(mg/g)	Mean	STD	CV
Cyanidin-3-O-rutinoside	Level 1	0.0024-0.0025	0.0024	3.8485e-05	0.0158
	Level 2	0.0028-0.0030	0.0029	4.2767e-05	0.0147
	Level 3	0.0035-0.0038	0.0037	7.1816e-05	0.0197
	Level 4	0.0032-0.0035	0.0034	6.9528e-05	0.0204
	Level 5	0.0039-0.0043	0.0041	1.0672e-04	0.0260
Pelargonidin-3,5-O-diglucoside	Level 1	0.0024-0.0025	0.0025	4.1685e-05	0.0170
	Level 2	0.0028-0.0030	0.0030	3.9719e-05	0.0136
	Level 3	0.0036-0.0038	0.0037	6.8497e-05	0.0185
	Level 4	0.0033-0.0035	0.0034	5.5051e-05	0.0163
	Level 5	0.0039-0.0042	0.0041	8.1196e-05	0.0217
Pelargonidin-3-O-galactoside	Level 1	0.0015-0.0024	0.0020	2.8996e-04	0.1465
	Level 2	0.0015-0.0016	0.0016	1.9421e-05	0.0124
	Level 3	0.0017-0.0018	0.0017	3.3768e-05	0.0199
	Level 4	0.0016-0.0017	0.0016	1.8634e-05	0.0115
	Level 5	0.0020-0.0021	0.0020	3.8782e-05	0.0194
Peonidin-3-O-galactoside	Level 1	0.00061-0.00064	0.00063	7.4890e-06	0.0120
	Level 2	0.00061-0.00063	0.00062	8.0800e-06	0.0131
	Level 3	0.00058-0.00060	0.00059	5.2343e-06	0.0088
	Level 4	0.00062-0.00065	0.00064	7.3966e-06	0.0117
	Level 5	0.00076-0.00081	0.00078	1.3745e-05	0.0175
Peonidin-3-O-glucoside	Level 1	0.00043-0.00046	0.00044	5.8985e-06	0.0133
	Level 2	0.00043-0.00045	0.00044	5.9864e-06	0.0135
	Level 3	0.00041-0.00043	0.00043	3.8953e-06	0.0091
	Level 4	0.00046-0.00047	0.00046	4.7153e-06	0.0102
	Level 5	0.00055-0.00059	0.00057	9.7444e-06	0.0172
Malvidin-3-O-glucoside	Level 1	0.00100-0.00104	0.00102	1.1980e-05	0.0118
	Level 2	0.00097-0.00101	0.00100	1.2805e-05	0.0129
	Level 3	0.00092-0.00096	0.00094	1.0447e-05	0.0111
	Level 4	0.00095-0.00099	0.00097	1.1895e-05	0.0122
	Level 5	0.00120-0.00127	0.00124	2.0990e-05	0.0170
Delphinidin	Level 1	0.00096-0.00102	0.00100	1.2462e-05	0.0126
	Level 2	0.00093-0.00098	0.00095	1.3275e-05	0.0140
	Level 3	0.00088-0.00091	0.00089	8.4986e-06	0.0095
	Level 4	0.00087-0.00091	0.00089	1.0920e-05	0.0123
	Level 5	0.00111-0.00118	0.00115	2.0171e-05	0.0176
Pelargonidin	Level 1	0.00069-0.00072	0.00070	8.3219e-06	0.0118
	Level 2	0.00067-0.00069	0.00068	8.5358e-06	0.0126
	Level 3	0.00063-0.00067	0.00065	9.7139e-06	0.0149
	Level 4	0.00062-0.00064	0.00063	6.9913e-06	0.0110
	Level 5	0.00080-0.00085	0.00082	1.4828e-05	0.0180

Peonidin	Level 1	0.00107-0.00112	0.00109	1.2774e-05	0.0117
	Level 2	0.00102-0.00107	0.00105	1.6036e-05	0.0153
	Level 3	0.00096-0.00101	0.00098	1.1263e-05	0.0115
	Level 4	0.00097-0.00100	0.00099	1.1082e-05	0.0110
	Level 5	0.00125-0.00131	0.00128	2.1226e-05	0.0165
Malvidin	Level 1	0.0036-0.0038	0.0037	4.3183e-05	0.0117
	Level 2	0.0035-0.0037	0.0036	4.6512e-05	0.0130
	Level 3	0.0033-0.0034	0.0034	3.1935e-05	0.0095
	Level 4	0.0033-0.0034	0.0033	3.6705e-05	0.0110
	Level 5	0.0042-0.0045	0.0044	7.4994e-05	0.0172
